# Carotenoid coloration and coloration-linked gene expression in red tilapia (*Oreochromis sp.*) tissues

**DOI:** 10.1186/s12917-021-03006-5

**Published:** 2021-09-25

**Authors:** Khristina G. Judan Cruz, Ervee P. Landingin, Maureen B. Gajeton, Somar Israel D. Fernando, Kozo Watanabe

**Affiliations:** 1 Department of Biological Sciences, College of Science, Central Luzon State University, Nueva Ecija Science City of Munoz, Philippines; 2 Department of Civil and Environmental Engineering, Ehime University, Bunkyo-cho 3, Matsuyama, 790-8577 Japan; 3 Center for Marine Environmental Studies (CMES), Ehime University, Bunkyo-cho 2-5, Matsuyama, Ehime 790-8577 Japan

**Keywords:** Carotenoid level, Coloration, red tilapia, *csf1ra*, *Bcdo2*, *StAR*

## Abstract

**Background:**

Production, marketability and consumer preference of red tilapia often depends upon the intensity of coloration. Hence, new approaches to develop coloration are now geared to improve market acceptability and profit. This study evaluated the effects of carotenoid-rich diets on the phenotypic coloration, carotenoid level, weight gain and expression of coloration-linked genes in skin, fin and muscle tissues. Carotenoids were extracted from dried *Daucus carota* peel, *Ipomoea aquatica* leaves, and *Moringa oleifera* leaves. Eighty (80) size-14 fish were fed with carotenoid-rich treatments twice a day for 120 days. The phenotypic effect of the carotenoid extracts was measured through a color chart. Skin carotenoid level was measured through UV-vis spectrophotometer. *csf1ra, Bcdo2* and *StAR* expression analysis was done using qRT-PCR.

**Results:**

Treatments with carotenoid extracts yielded higher overall scores on phenotypic coloration and tissue carotenoid levels. Differential expression of carotenoid-linked genes such as the elevated expression in *csf1ra* and lower expression in *Bcdo2b* following supplementation of the enhanced diet supports the phenotypic redness and increased carotenoid values in red tilapia fed with *D. carota* peel and *I. aquatica* leaves.

**Conclusions:**

Overall improvement in the redness of the tilapia was achieved through the supplementation of carotenoid-rich diet derived from readily available plants. Differential expression of coloration-linked genes supports the increase in the intensity of phenotypic coloration and level of carotenoids in the tissues. The study emphasizes the importance of carotenoids in the commercial tilapia industry and highlights the potential of the plant extracts for integration and development of feeds for color enhancement in red tilapia.

## Background

The phenomenal growth of aquaculture production has pushed researches to improve the quality of the fish in the past three decades. Among the most cultured fish, the tilapia (*Oreochromis sp.*) stands out as one of the most important, possessing remarkable characteristics for breeding, disease resilience and faster growth [1, 2]. Their popularity paved for species genetic improvement that produced superior strains with enhanced performance such as higher productivity and survival [3]. Among the genetically enhanced strains, the red tilapia is considerably preferred than that of the other tilapia hybrids [4, 5] due to its attractive color and bright pigmentation. This strain was produced from a mutant cross of up to four different species, largely dominated by *Oreochromis mossambicus* and *O. niloticus* species [6]. Since its marketability is highly influenced by its color, several researches are now geared towards improving red tilapia pigmentation to enhance color quality and profit.

It is widely recognized that consumers subconsciously associate coloration with quality, flavor, nutritive value and general acceptability [7–10] which often impacts commercial value, especially in fishes [7, 8]. Significant work has demonstrated that coloration in fishes is highly influenced by the carotenoid level in their diets [9, 11]. To date, dietary supplementation of carotenoids in aquaculture have been used extensively and feed formulation have been gaining considerable interest especially for fish cultured for human consumption, particularly Nile tilapia, as a result of market demand and preference to colorful fishes [12, 13].

The role of carotenoids in fish health is widely recognized. Carotenoids does not only provide consistent pigmentation [11, 14] but also affect fish growth, performance and overall health [15] affecting key production parameters in fish. Carotenoids serve functions in cellular pathways [16] that increase the metabolism of animals [15]. Carotenoids are one of the most powerful antioxidants [16] which provides protection against several stressors including ultraviolet (UV) radiation, reactive oxygen species and free radicals [15, 17]. Carotenoids also serve as precursors of transcription regulators and plays a critical role in the immune system [15, 18–21]. It has also been shown that fishes with elevated carotenoid levels are more resistant to bacterial and fungal diseases [7, 8, 10]. Carotenoids, thus, are routinely added to the diets not only for pigmentation, but also for their optimal health.

Since fishes cannot synthesize carotenoids de novo [7, 8, 11, 22], suitable feeds must be formulated through natural carotenoid supplementation incorporated in fish meals to efficiently provide the carotenoids necessary for the target color enhancement and intensification [7, 8, 15, 23–25]. Naturally-occurring carotenoids such as those in carotenoid-rich plants can be tapped as sources for feed supplementation for color enhancement. Natural carotenoids are generally safer than synthetic carotenoids [7, 8, 22] typically incorporated in fish feeds by commercial fish producers for faster color intensification and maintaining overall fish health. Integrating natural carotenoids is a more practical approach in aquaculture since these plant materials are readily available, easily incorporated in the fishmeal and usually freely.

As to date, only the study of Velasco et al. [26] reports on the effect of carotenoid supplementation on the general phenotypic coloration in red tilapia. This paper further explored the effect of carotenoid-rich diet formulated from three plants not only on the general coloration but also on the carotenoid level and molecular expression in the integument, muscle and fin. The understanding on the pathways behind carotenoid regulation is relatively limited [16], especially on the genes regulating its metabolism [14]. Genes linked to coloration expression, such as *csf1ra* [13]*, Bcdo2b* [27] and *stAR* [28], were included in this study to evaluate the effect of carotenoids at the molecular level.

## Results

### Phenotypic color measurement in the fish

**Fig. 1:**
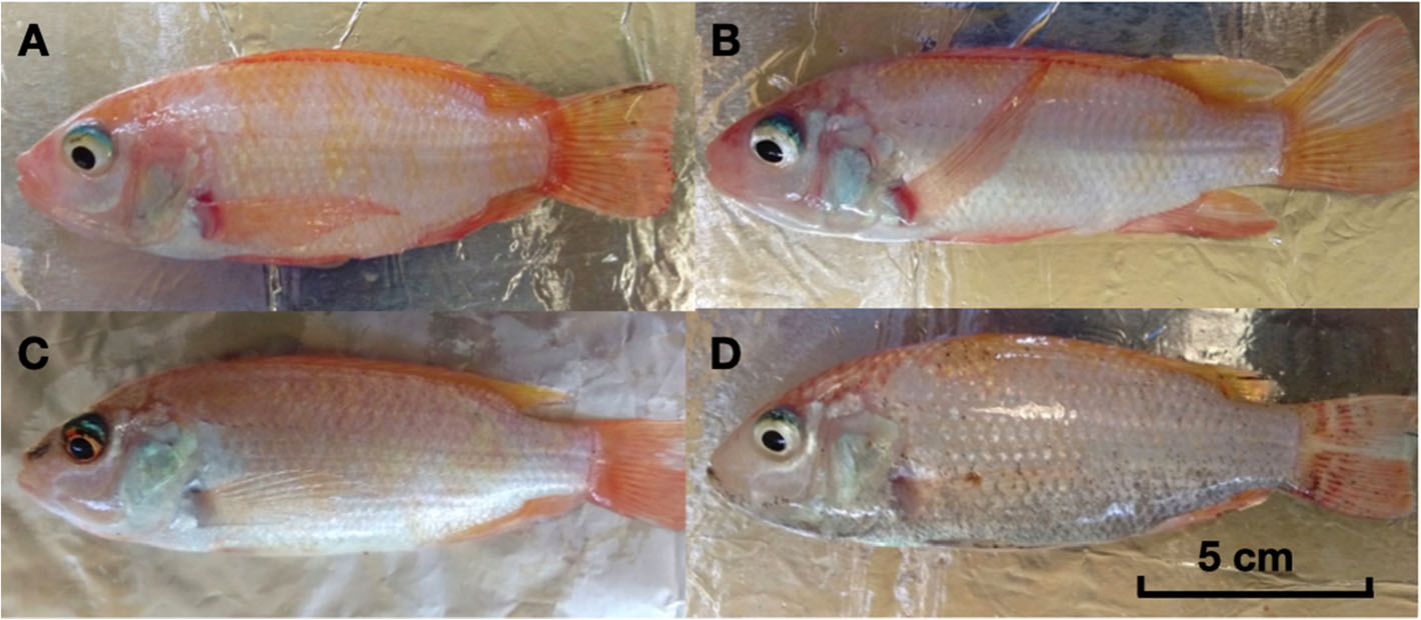
Red Tilapia samples as affected by different treatments (A = *D. carota*; B = *I.* *aquatica*; C = *M. oleifera* and D = control) after the treatment period. Reference measurement in cm

Treatments with *D. carota* extracts yielded higher overall scores on phenotypic coloration than that fed with control feeds (pure commercial feeds) (Table 1**;** values are presented as mean ± standard deviation). The phenotypic redness in skin, fin and muscle coloration is highly observed in *Daucus carota* peel extracts with mean values significantly elevated than control feeds in all the tissues observed. *Ipomoea aquatica* and *Moringa oleifera* leaf extracts also obtained statistically higher values than the control. Figure 1 shows the differences in phenotypic color of the samples after the treatment period.

**Table 1:** Phenotypic coloration scores on the effect of carotenoid-rich extracts in red tilapia

TREATMENT	SKIN	FIN	MUSCLE
*D. carota* peel+ feeds	6.25 ± 1.37^a^	9.35 ± 1.14^a^	5.25 ± 1.07^a^
*I. aquatica* leaves+ feeds	4.60 ± 0.75^b^	8.10 ± 1.29^b^	4.00 ± 1.56^b^
*M. oleifera* leaves+ feeds	3.80 ± 1.06^b^	6.60 ± 0.75^c^	4.05 ± 1.10^b^
Control Feeds	2.05 ± 0.76^c^	4.00 ± 0.45^d^	2.45 ± 0.60^c^
*P*	<.001	<.001	<.001

Note: Comparison in columns. Values are presented as mean ± standard deviation. Means within the same column with same superscript letter are not significantly different

### Carotenoid level measurement of tissues

Consistently, *D. carota* treatments showed significantly higher levels of carotenoids in all tissues (Table 2**;** values are presented as mean ± standard deviation). *I. aquatica* and *M. oleifera* were also observed to have higher carotenoid values in skin, fins and muscles compared to the control (pure commercial feeds), even though the values are slightly lower than *D. carota*. Carotenoid level values in fins showed significant differences between the treatments of *I. aquatica* and *M. oleifera*.

**Table 2:** Carotenoid values in different tissues of the red tilapia as affected by the different treatments

TREATMENTS	SKIN	FINS	MUSCLE
*D. carota* peel+ feeds	2.18 ± 0.13^a^	2.82 ± 0.13^a^	1.68 ± 0.15^a^
*I. aquatica* leaves+ feeds	1.54 ± 0.12^b^	1.90 ± 0.13^b^	1.53 ± 0.06^a^
*M. oleifera* leaves+ feeds	1.41 ± 0.06^b^	1.23 ± 0.10^c^	1.50 ± 0.21^a^
Control Feeds	0.58 ± 0.14^c^	0.70 ± 0.13^d^	0.53 ± 0.02^b^
*P*	<.001	<.001	<.001

Note: Comparison in columns. Values are presented as mean ± standard deviation. Means within the same column with same superscript letter are not significantly different

### Effect of carotenoid-rich diet on the weight gain of red tilapia

For weight gained after the treatment period, treatments with *D. carota* and *I. aquatica* have significantly higher values with averages of 4.91 g and 5.17 g as compared to the control (4.89 g) and *M. oleifera* (4.75 g) treatments.

### Relative expression of c*sf1ra*, *Bcdo2b* and *StAR*

**Fig. 2:**
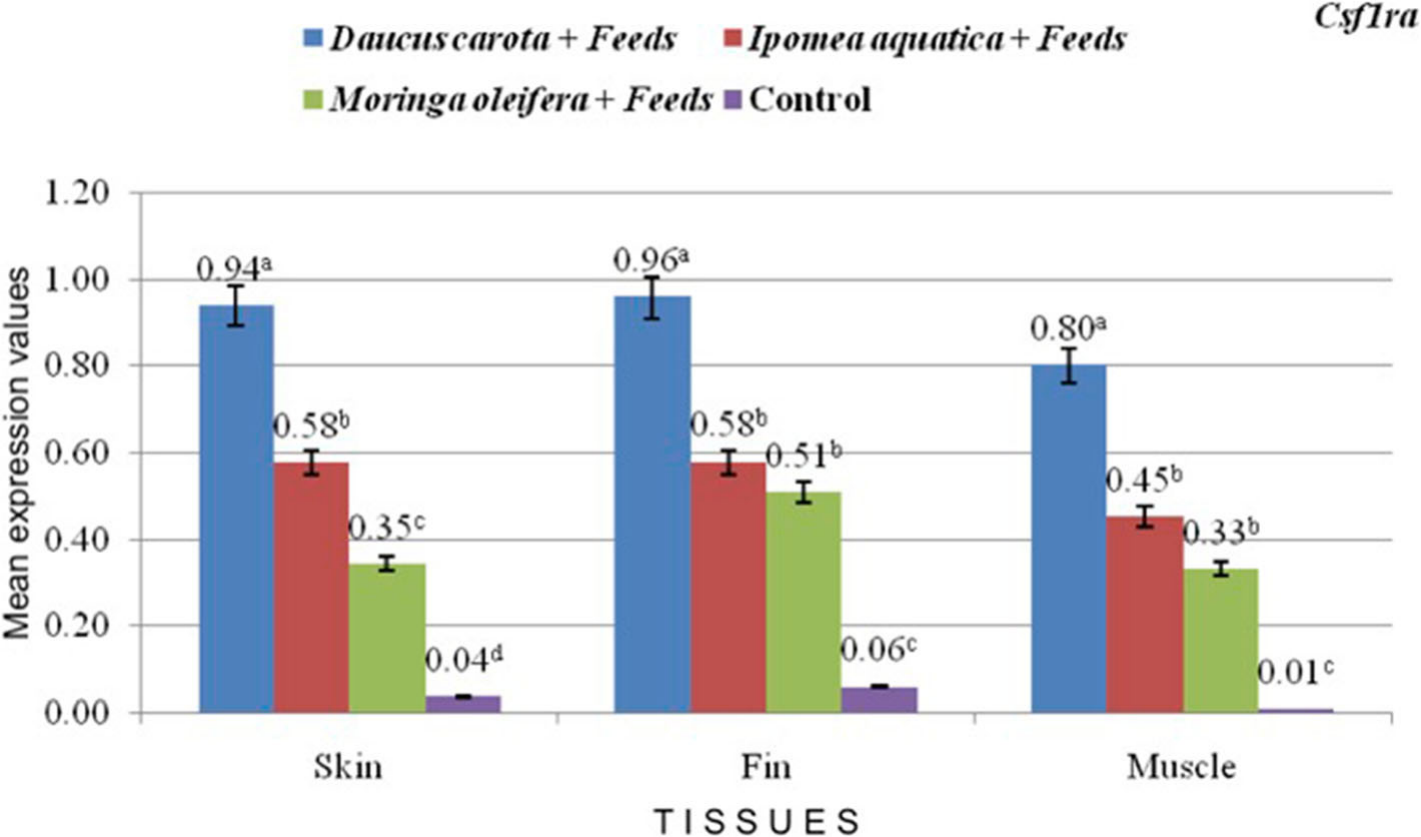
mRNA expression of *Csfr1a* in the skin, fins, and muscle of red tilapia as affected by the different treatments

**Fig. 3:**
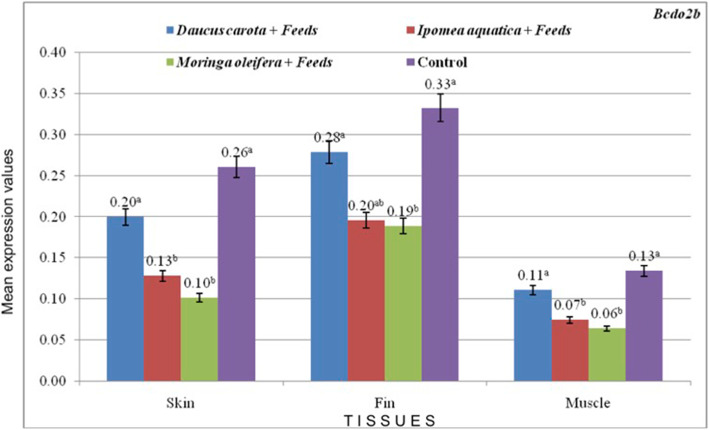
mRNA expression of *Bcdo2b* in the skin, fins, and muscle of red tilapia as affected by the different treatments

**Fig. 4:**
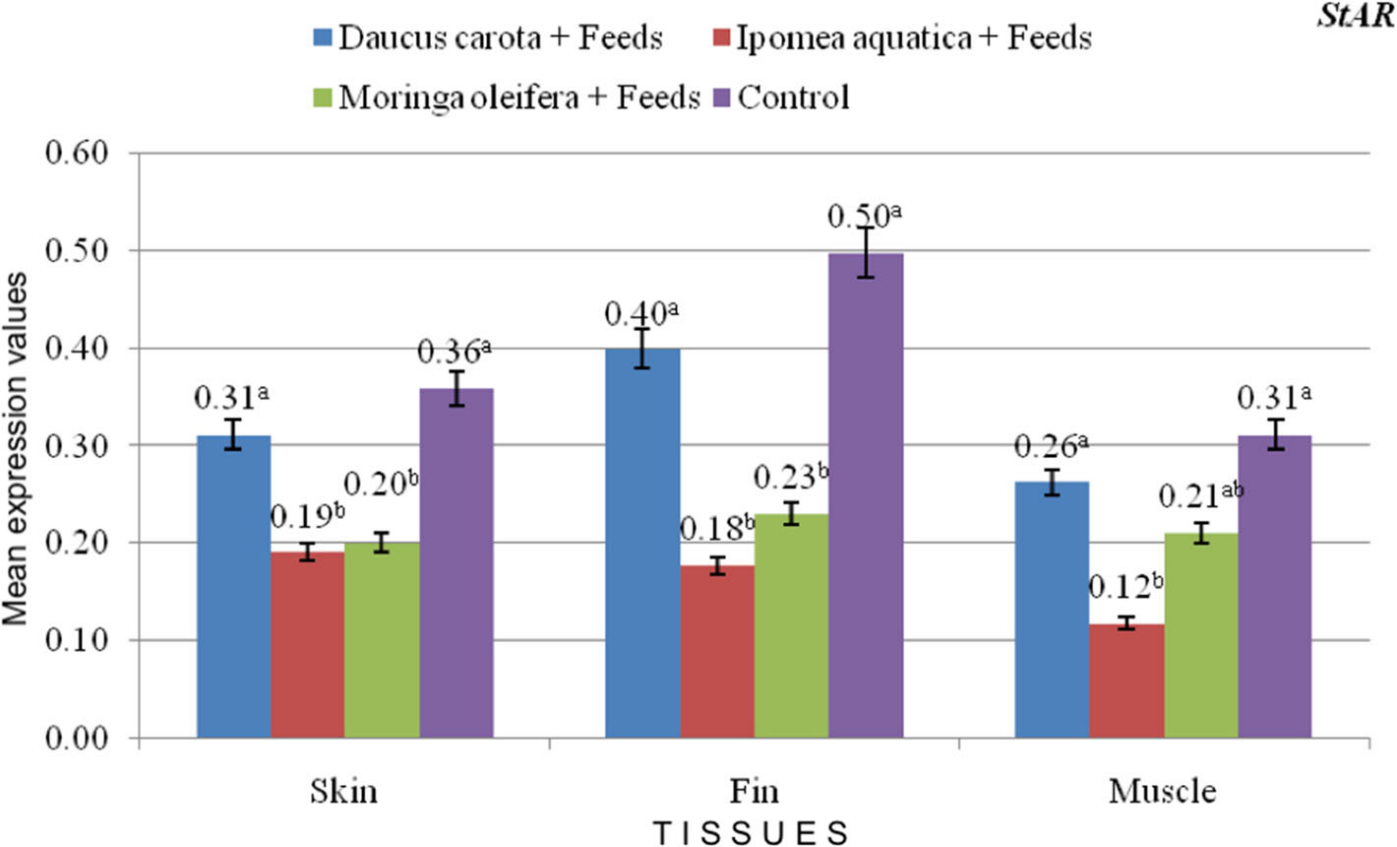
mRNA expression of StAR Bcdo2b in the skin, fins, and muscle of red tilapia as affected by the different treatments

The expression of *csf1ra* significantly increased in skin, fin and muscle tissues as affected by the carotenoid-rich treatments compared to the control feeds (Fig. 2**;** values are presented as mean) and is parallel to the results of the phenotypic redness and carotenoid values of the treatments. Significantly higher expression of *csf1ra* among treatments may indicate carotenoid-uptake in the test samples. Among the plants tested, *D. carota* treatment had the highest expression of *csf1ra* in skin, fin and muscle tissues. Higher numerical values also indicate prominence of *csf1ra* expression in the fin tissues. Since *Bcdo2b* is responsible for white coloration*,* it registered lower expression values in the treatments (Fig. 3**;** values are presented as mean). Expression of *StAR* in carotenoid treatments have insignificant differences with control feeds with each tissue evaluated **(**Fig. 4**;** values are presented as mean**)**.

## Discussion

The more prominent coloration in both skin and fins in all carotenoid treatments compared to the muscles may be attributed to the fact that concentration of carotenoids in fishes mostly occurs in their integument [7, 8, 11, 15] which mainly influence their coloration [13]. This is particularly true in cichlids where build-up of carotenoids has been apparent following dietary supplementation of carotenoids [13]. Concentration of carotenoid uptake in muscles maybe observed in other groups of fishes such as the Salmonidae in the form of astaxanthin [11].

The carotenoid values support findings in the scores of the phenotypic color evaluation and may indicate uptake of carotenoids among the samples. Among the plants evaluated, *D. carota* showed higher scores in phenotypic redness and increased levels of carotenoids in the tissues evaluated following diet supplementation. *D. carota* contain the highest amount of carotenoids among the plant treatments [29]. Albeit, all plants tested contain an important carotenoid, the β-carotene, which may enhance general coloration upon consumption. *D. carota* contains 16–38 mg/100 g of β-carotene while *I. aquatica* and *M. oleifera* have 18–34 mg/100 g and 17.4 mg/100 g, respectively [30–32].

Similar results were observed in the study of Velasco et al. [26] where different red tilapia strains fed with carotenoid extracts showed enhanced growth, pigmentation and survival rate. *D. carota* contains fiber, vitamin K1, potassium, and antioxidant [33] that aids in improving health in *O. niloticus. I. aquatica* is a good source of natural antioxidants, minerals, protein, vitamin C, calcium and potassium [30]. These various types of antioxidant compounds include ascorbic acid, flavonoids, phenolics and other carotenoids [34, 35]. Nutritional studies reported carotenoid functions in fish that range from general enhancement of performance to reproductive and metabolic functions [36]. A type of carotenoid, astaxanthin, is essential for growth and survival of fish [36]. The effect in weight by carotenoid supplementation maybe fish-dependent or possibly affected by several factors. Contradicting results were seen by Rahman et al. [37] wherein the use of astaxanthin revealed no significant differences in survival, weight gain, specific growth rate, feed efficiency, daily feed intake, daily protein intake, and protein efficiency ratio even with the increase in pigmentation in *Oncorhynchus mykiss*. Their results were also consistent in other commercial fishes such as rainbow trout, Atlantic salmon, characins (*Hyphessobrycon callistus*), red porgy (*Pagrus pagrus*) and gilthead seabream (*Sparus aurata*) [37]. Since this study only involved 120 days of feeding, an extended period of supplementation might be necessary to verify the effect of carotenoids on growth performance [38].

The colony-stimulating factor 1 receptor a or *csf1ra* is an important marker of pigment pattern formation and xanthopore development [39, 40] involved in carotenoid-containing integument in cichlids [13] that is responsible for the expression of yellow to red coloration. The expression of *csf1ra* in yellow-colored areas in cichlids is related to xanthophore recruitment [13, 39] wherein carotenoids are stored [13, 39, 41, 42]. Xanthophores appear to have supplemental biochemical pathways enabling individuals to accumulate yellow pigments from external factors [43] and play a role in the pigment pattern formation and skin coloration especially when migrating to the outer skin or epidermis layer [44–46]. Thus, elevated expression of the *csf1ra* may connote red to yellow skin coloration in fish. Lower expression of *csf1ra* in the red tilapia skin fed with *M. oleifera* leaves could be attributed to its comparable phenotypic color with the control.

*Bcdo2b* (β, β-carotene-9′,10′-dioxygenase 2) functions as a cleaving enzyme that cleaves colorful β-carotenoids into colorless apocarotenoids [27], as observed in the avian species [47]. As such, *Bcdo2* is responsible for white skin coloration. It acts as a carotenoid scavenger and gatekeeper for the mitochondrial apoptotic pathway in which it functions as a carotenoid catabolite in zebrafish [48]. The loss of function *Bcdo2b* also aids in the increased levels of β-carotene [49, 50]. Hence, treatments with improved redness coloration showed lower expression of *Bcdo2b*. The low expression values of *Bcdo2b* (Fig. 1) and the significantly higher expression values of *csf1ra* may indicate the uptake of carotenoids, hence, the enhanced yellow to red coloration in the skin of the red tilapia in the treatments.

Although the Steroidogenic acute regulator (*StAR*) gene is a candidate gene for carotenoid coloration in red-billed quelea and categorized as a site of deposition and binding of carotenoids [28], another carotenoid-binding and uptake related gene may have been expressed such as SR-B1, MLN64, gsta2 or PLIN2 [28, 40] or *StAR* may have been expressed in other tissues such as the intestine, where carotenoids are mostly transported, and gonads, where carotenoids are also stored in fishes. Moreover, relatively lower expression may be attributed to the fact that *StAR* responds highly to colorless molecules like cholesterols and fats [28]. *StAR* is a transport protein that regulates the transfer of cholesterol within the mitochondria.

General enhancement in the redness of tilapia was attained through the supplementation of carotenoid-rich diet derived from readily available plants. Differential expression of carotenoid-related genes such as the elevated expression in *csf1ra* and lower expression in *Bcdo2b* following supplementation of enhanced diet support the phenotypic redness and increased carotenoid values in red tilapia fed with *D. carota* peel and *I. aquatica* leaves.

Much remains to be understood on the underlying molecular mechanisms controlling the uptake and effect of carotenoid-rich supplementation. Although genes involved in carotenoid processes such as metabolism are relatively limited, several genes may be evaluated to determine the effects of this diet on the coloration in red tilapia as well as on the metabolism and storage of carotenoids in carotenoid-rich regions. These may include the transition factor *Pax3*, receptor tyrosine kinase *csf1r*, genes *Edn3b*, *dgat2, bscl2, faxdc2, retsatl, CYP2J19* and *SCARB1* [14, 39, 42, 51–53]. Studies on these genes may give crucial insights to potential carotenoid candidate genes in tilapia. Indeed, genetic factors constitute an essential role in the understanding of carotenoid-based coloration.

Further experiments are needed for an in-depth evaluation on the carotenoid uptake of these plants in tilapia. Nevertheless, this study showed that carotenoid-rich diet significantly improves phenotypic coloration and carotenoid levels in red tilapia and emphasizes the importance of carotenoids in the commercial fish industry. The results are particularly significant in tilapia culture. The intensity of the fish color plays an important marketing strategy as it is subconsciously associated with nutritional value which influences higher demand and consumption. Carotenoid supplementation does not only contribute to improving quality by enhancing color, but also improves growth rates and reproductive performance. This, in turn, highly boosts production and profits. Therefore, considerable potential is shown on the use of natural plant-based carotenoids in aquaculture feed industries and fish farming.

## Conclusions

Overall improvement in the redness of red tilapia was achieved through the supplementation of carotenoid-rich diet. Differential expression of coloration-linked genes supports the increase in the intensity of phenotypic coloration and level of carotenoids in the tissues. The study emphasizes the importance of carotenoids in the commercial tilapia industry and highlights the potential of the plant extracts for integration and development of feeds for color enhancement in red tilapia.

## Methods

### Collection and preparation of extracts and experimental diets

Samples of *D. carota* were obtained from the Cordillera Administrative Region (Baguio), Philippines. *I. aquatica* leaves and *M. oleifera* leaves were collected in the Science City of Munoz, province of Nueva Ecija (Philippines) by Ms. Ervee P. Landingin. Voucher specimens were identified by Mr. Paul Henric P. Gojo Cruz (Department of Biological Sciences, Central Luzon State University). *D. carota* peel, *I. aquatica* leaves and *M. oleifera* leaves were dried in an oven with temperature of 50 °C for about 8 h until the plant samples were completely dried and suitable for grinding. The dried plant samples were pulverized and sieved to get fine particles. Acetone was used to extract carotenoid pigments. One hundred fifty grams (150 g) of dried plant samples was immersed individually in 750 ml of acetone for 24 h. The extracts were filtered, air dried and kept refrigerated until use. Five hundred milligrams (500 mg) of the collected pigment extract were dissolved in 500 ml of ethyl alcohol separately and mixed in one kilogram of commercial feed. The prepared diets were air-dried until the alcohol completely evaporated and were kept in container until use. This method was adapted from Velasco et al. [26]. The carotenoid concentration was computed using the formula of de Carvalho et al. [54] that resulted to 500 mg/kg ratio of extracts to feeds. This concentration is considered high in comparison to the carotenoid concentrations in commercial formulated fish diets reported by Wallat et al. [55].

Pure commercial fish feed (BMEG Premium Fry Mash, San Miguel Corp) was used as control with the following components in percent (%): crude protein (31), crude fiber (7), crude fat (9), ash (16) and moisture (13). The commercial feed was mixed with respective carotenoid extracts for the treatments with carotenoid-rich diet.

### Fish and treatment set-up

The red tilapia samples were reared at the Freshwater Aquaculture Center, Central Luzon State University, Nueva Ecija (Philippines). Eighty (80) size-14 red tilapia weighing 4–5 g were stocked randomly and separately in fifteen-liter capacity aquaria measuring 10 in. × 10 in. × 10 in. holding 12 l of water. The fish were acclimatized for 2 weeks prior to set-up. The treatments were named after the plant extracts received: *D. carota*, *M. oleifera* and *I. aquatica*. A total of 80 fish consisting 4 runs with 5 fish per treatment were used for analysis.

Aquaria were regularly maintained to ensure cleanliness and prevent water quality deterioration. Aerators were installed. Water parameters such as temperature and dissolved oxygen were monitored. The fish were monitored and fed twice daily using the feeds mixed with carotenoid-rich plant extracts. Feeding rate was ad libitum for the duration of 120 days. Initial and final weights of the fish were determined.

Fish were handled and sacrificed for the collection of tissues following the Institutional Animal Care and Use Committee (IACUC) policies, procedures and guidelines, with institutional approval from the Central Luzon State University (Philippines).

### Color measurement of the fish

**Fig. 5:**
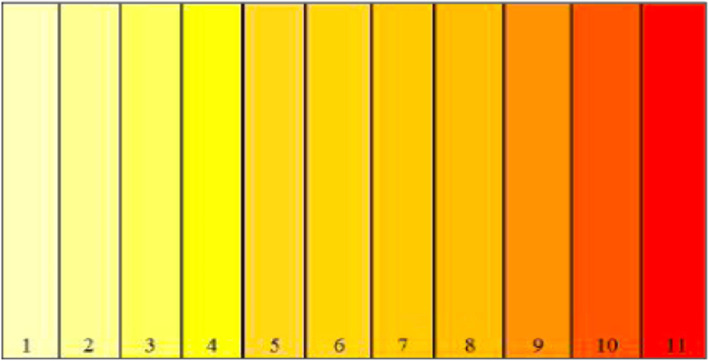
Color chart with assigned values for phenotypic coloration evaluation as developed by Velasco et al. [25]

The experimental fish were photographed using a digital camera. A color chart developed by Velasco et al. [26] (Fig. 5) with corresponding degrees of coloration from light yellow to red orange and assigned values (1–11) was used in differentiating the degree of skin coloration in all treatments.

### Measurement of carotenoids

Prior to collection of tissues, fish were euthanized with buffered MS-222 with a concentration of 250 mg/L and pithed with a 1-cc syringe needle following the 2-step method for the sacrifice of cichlids. 150 g of skin, fin and muscle tissues of the experimental fish with different treatments were collected after 120 days of feeding and were placed in falcon tubes with 2 mL of acetone. Samples were stored at − 20 °C before analysis. The samples were placed in room temperature to allow the acetone to evaporate overnight. Leftover liquid in the sample (2 μL) was used in the measurement of the carotenoid where the absorbance of the extracts was determined at 449 nm wavelength in a UV-vis spectrophotometer [56].

### Gene expression analysis

#### RNA extraction

The tissue samples were lysed and homogenized in TRIzol™ Reagent by adding 500 μL of TRIzol™ Reagent per 50–100 mg of tissue to the sample and were homogenized using a homogenizer. This was incubated for 5 min to permit complete dissociation of the nucleoproteins complex. Chloroform, 100 μL, was added per 500 μL of TRIzol™ Reagent used for lysis, and then the tube was securely capped. This was incubated for 2–3 min at room temperature. The samples were centrifuged for 15 min at 12,000 x g at 4 °C. The mixture was separated into lower red phenol chloroform, and interphase, and a colorless upper aqueous phase. The aqueous phase, which contains RNA, was then transferred to a new tube by angling the tube at 45° and the solution was pipetted out. Isopropanol of 250 μL amount was added to the aqueous phase per 500 μL of TRIzol™ Reagent used for lysis. This was incubated at room temperature for 10 min then was centrifuged for another 10 min at 12,000 x g at 4 °C. The supernatant was discarded with a micropipettor. The pellet was resuspended in 500 μL of 75% ethanol per 500 μL of TRIzol™ Reagent used for lysis. The samples were vortexed then were centrifuged for 5 min at 7500 x g at 4 °C. The supernatant was discarded with a micropipettor. The RNA pellet was vacuumed or air dried for 5–10 min. The pellet was resuspended in 20–50 μL of RNase-free water, 0.1 mM EDTA, or 0.5% SDS solution by pipetting up and down. This was incubated in a water bath or heat block set at 55–60 °C for 10–15 min. The RNA was stored at − 80 °C.

#### Quantitative RT-PCR

A total of 80 RNA samples from the different treatments were subjected to gene expression analysis. Reactions were done through qRT-PCR analysis in a total volume of 10 μL solution containing 1 μL of the RNA template, 5 μL of 2x KAPA FAST SYBR Kit (KAPA Biosystems, USA), 0.2 μL RT mix, 0.5 μL each of the 10 μM forward and reverse primers and 2.8 μL of Diethylpyrocarbonate- (DEPC-) treated water (Invitrogen, USA). β-actin served as the internal standard. The following conditions were used: initial hold 42 °C 5 min, hold at 95 °C 2 mins and 45 cycles of 95 °C for 20 s; 60 °C for 20 s; 72 °C for 20 s. Final extension at 72 °C for 10 min. The primers used were the following: internal standard *β-actin*F 5′-GCTACTCCTTCACCACCACAG-3′, *β-actin*R 5′-CGTCAGGCAGCTCGTAACTC-3′ [57]; *csf1ra*F 5′-AACTGGAGGAGGAGCAGGTAATC-3′, *csf1ra*R 5′-GTGACACTTAGGCTTGTCATACG-3 ′[58]; *Bcdo2b*F 5′-CCCCAGAGCCCATTACGA-3′, *Bcdo2b*R 5′- TTTCAAGTGTTTCTGGATC-3′ [48]; *stAR*F 5′-ACCCCTCTGCTCAGGCATTT-3′, *stAR*R 5′-GGGCTCCACCTGCTTCTTG-3′ [59]. Amplification was done using Bio-Rad CFX96TM Real-Time thermal cycler.

#### Statistical analysis

For the phenotypic color measurement, Kruskal-Wallis H test was used to determine the significant differences between the groups followed by Wilcoxon-Mann Whitney test for the comparison of means. General linear model univariate analysis (SPSS v16) was used for the analysis of the carotenoid level. To calculate the relative gene expression of the samples, the 2–∆∆Ct (Livak) method [60] was used. The mRNA expression values were analyzed using one-way Analysis of Variance (ANOVA) (SPSS v16) in a completely randomized design (CRD) followed by Tukey’s Honest Significant Difference test (HSD) for the comparison of means. The linear additive model for the CRD is:

*Y*_*ij*_ = *μ + τ*_*i*_ *+ ε*_*ij i =* 1,2,3,4, and *j =* 1,2…,20_.

*Y*_*ij*_ is the j^th^ score of the i^th^ treatment.

*μ* is the overall mean effect.

*τ*_*i*_ is the treatment effect of the i^th^ treatment.

*ε*_*ij*_ is the random error.

## Data Availability

All data generated or analyzed in this study are available from the corresponding author on reasonable request.

## Abbreviations

csf1ra: Colony-stimulating factor 1 receptor a
Bcdo2: β, β-carotene-9’,10’-dioxygenase 2
StAR: Steroidogenic acute regulator
RNA: Ribonucleic acid
qRT-PCR: Real-time quantitative reverse transcription polymerase chain reaction
Ct: threshold cycle
